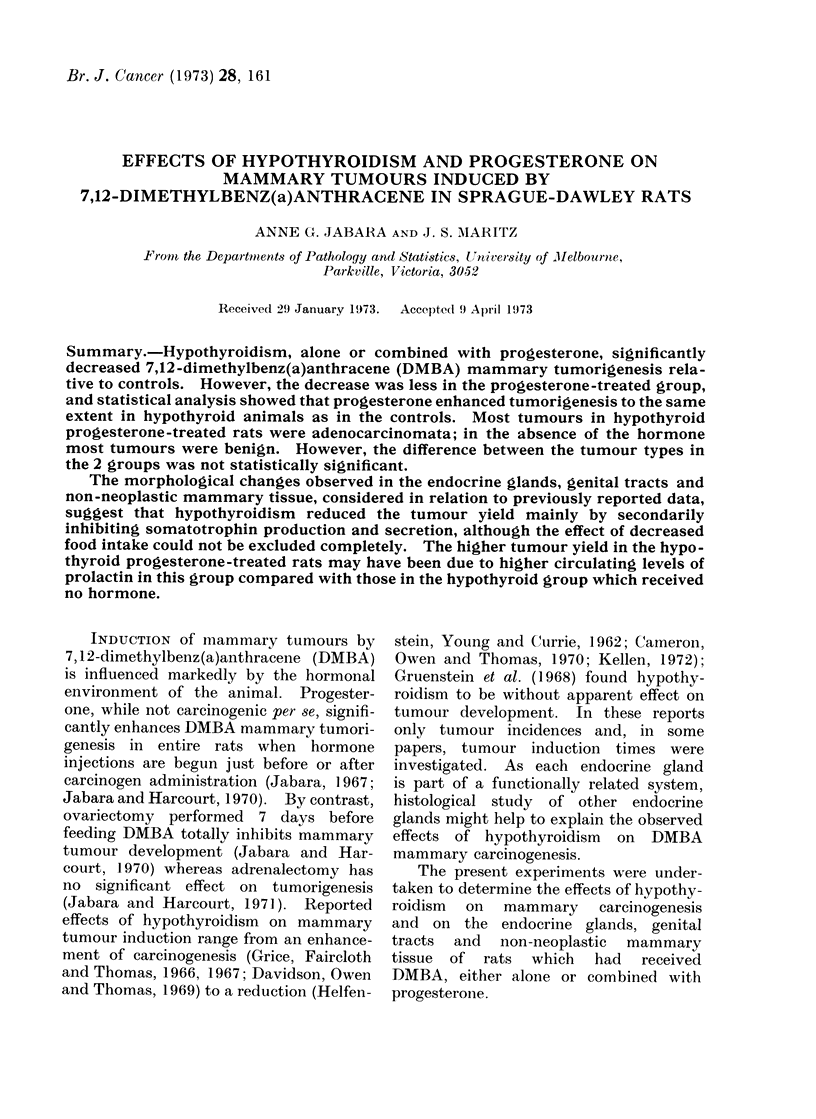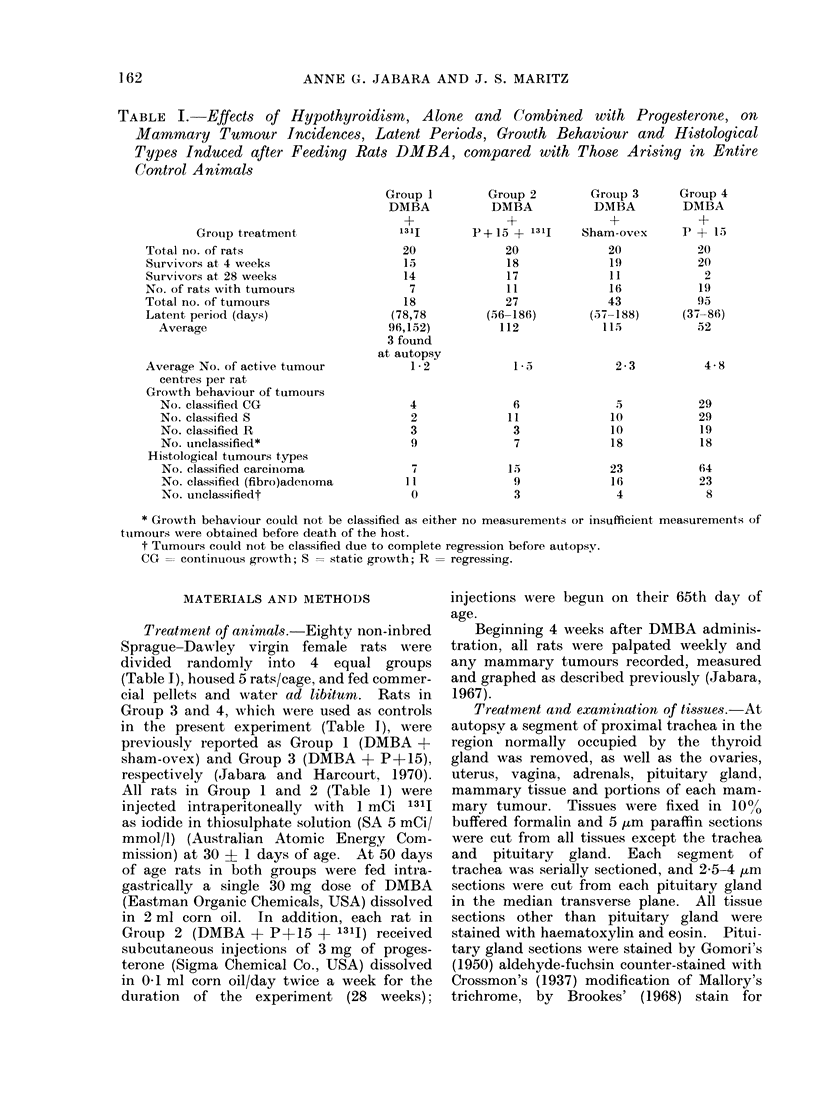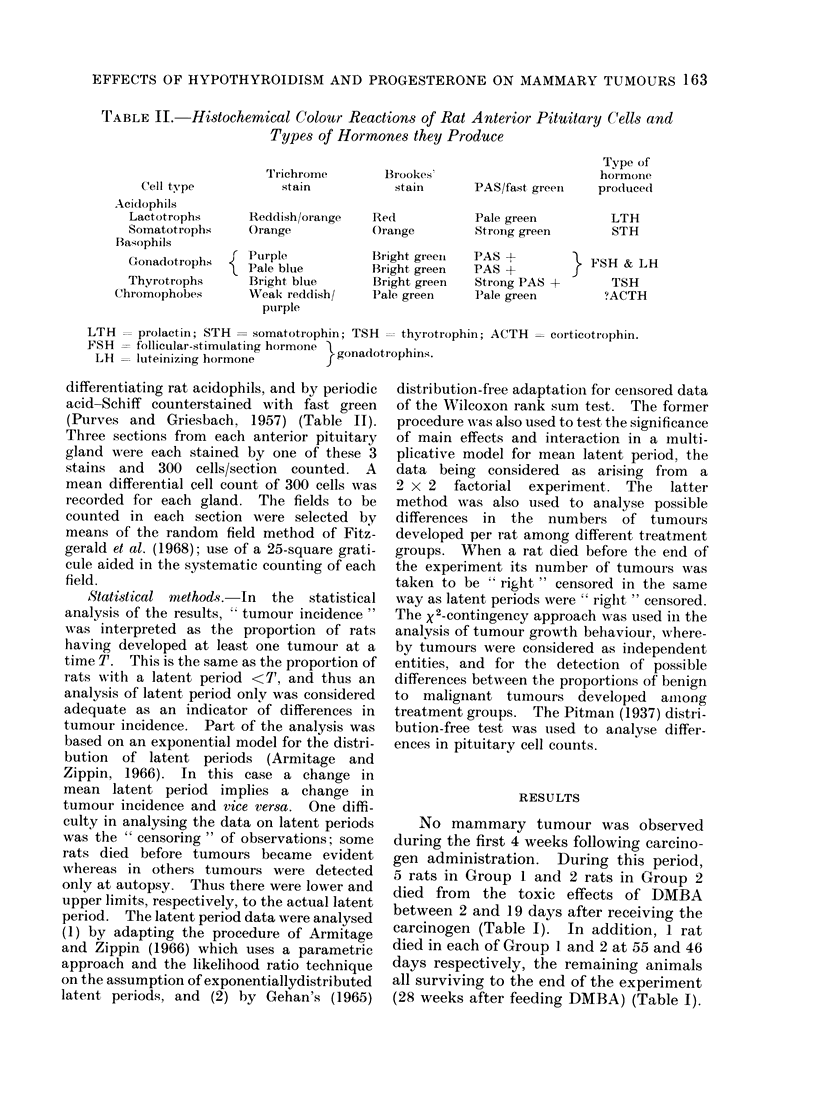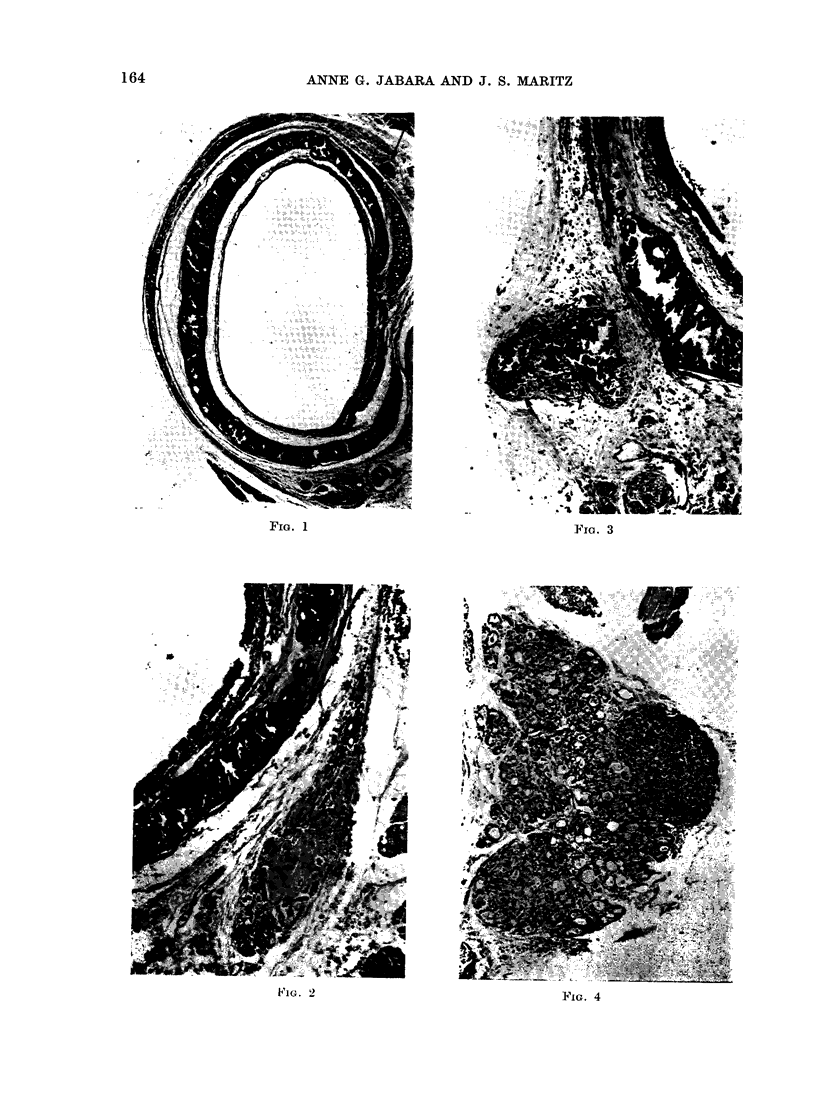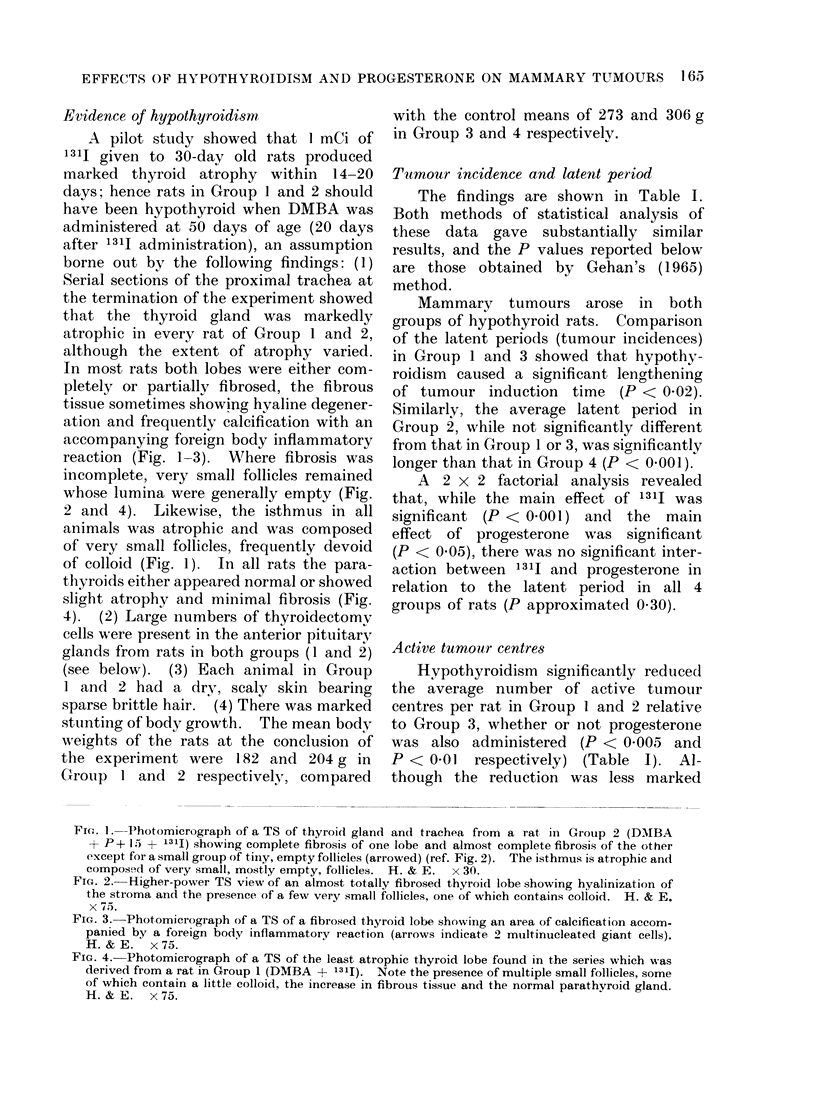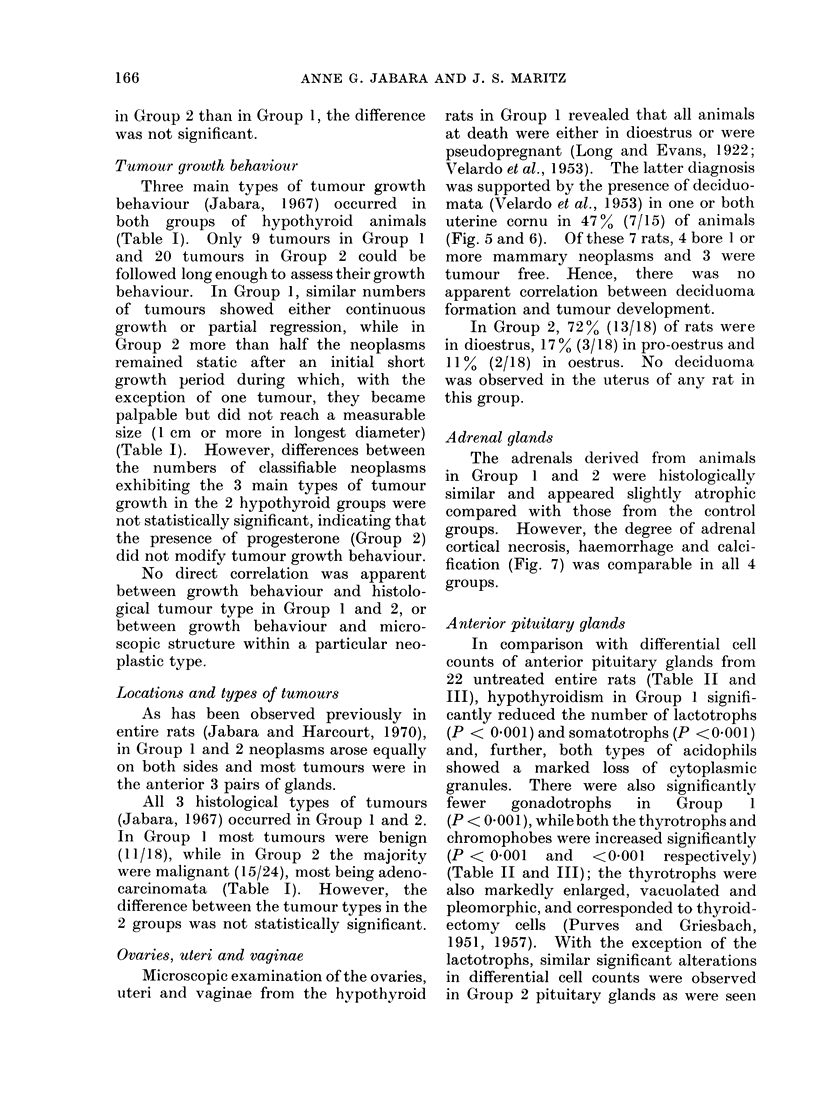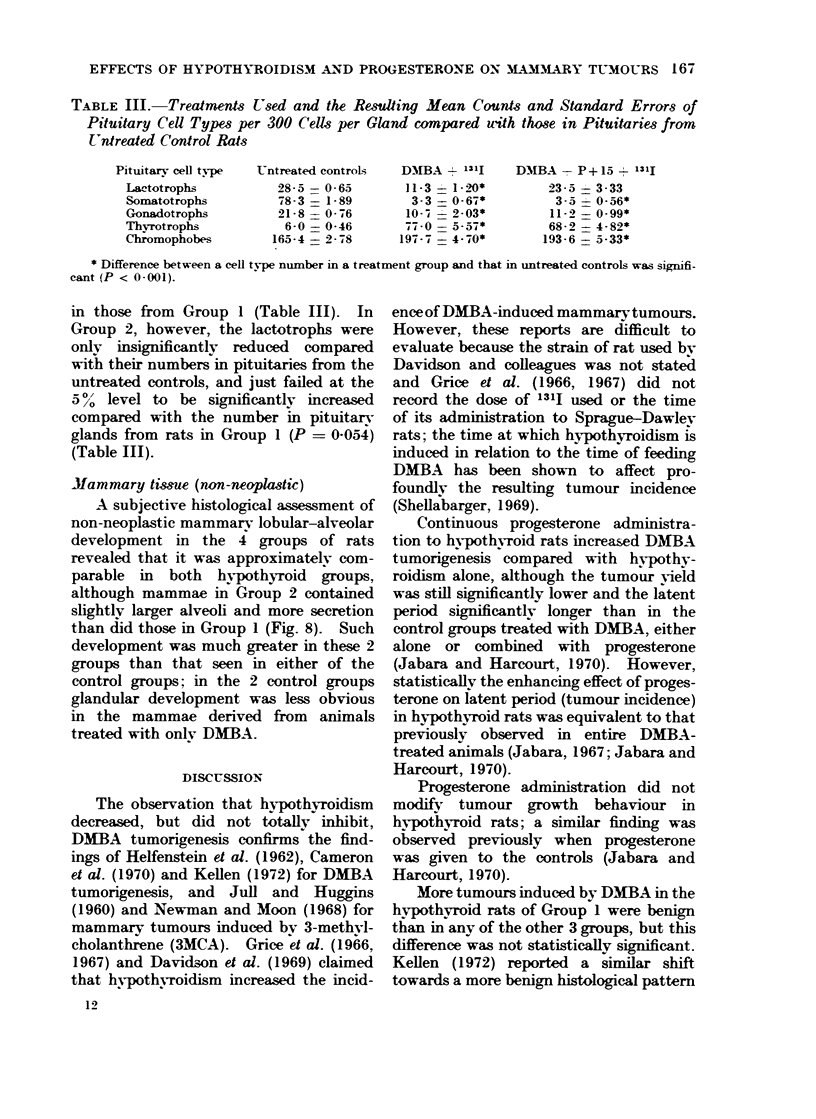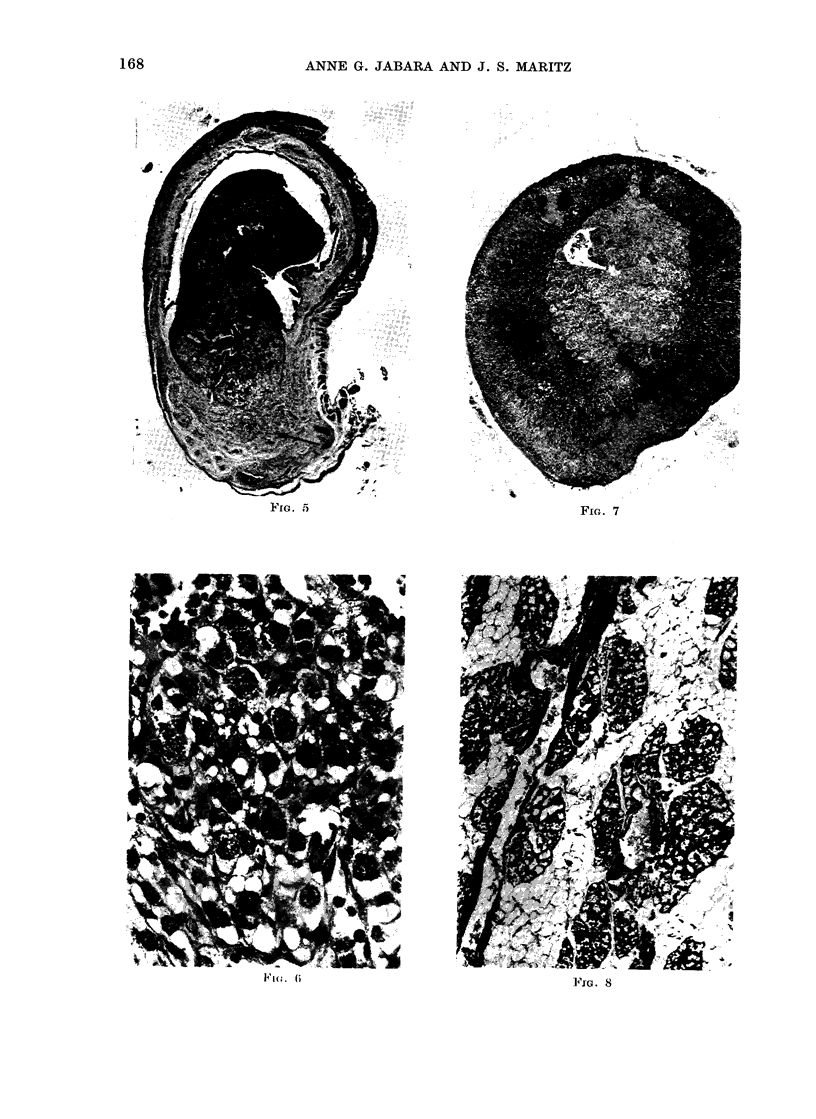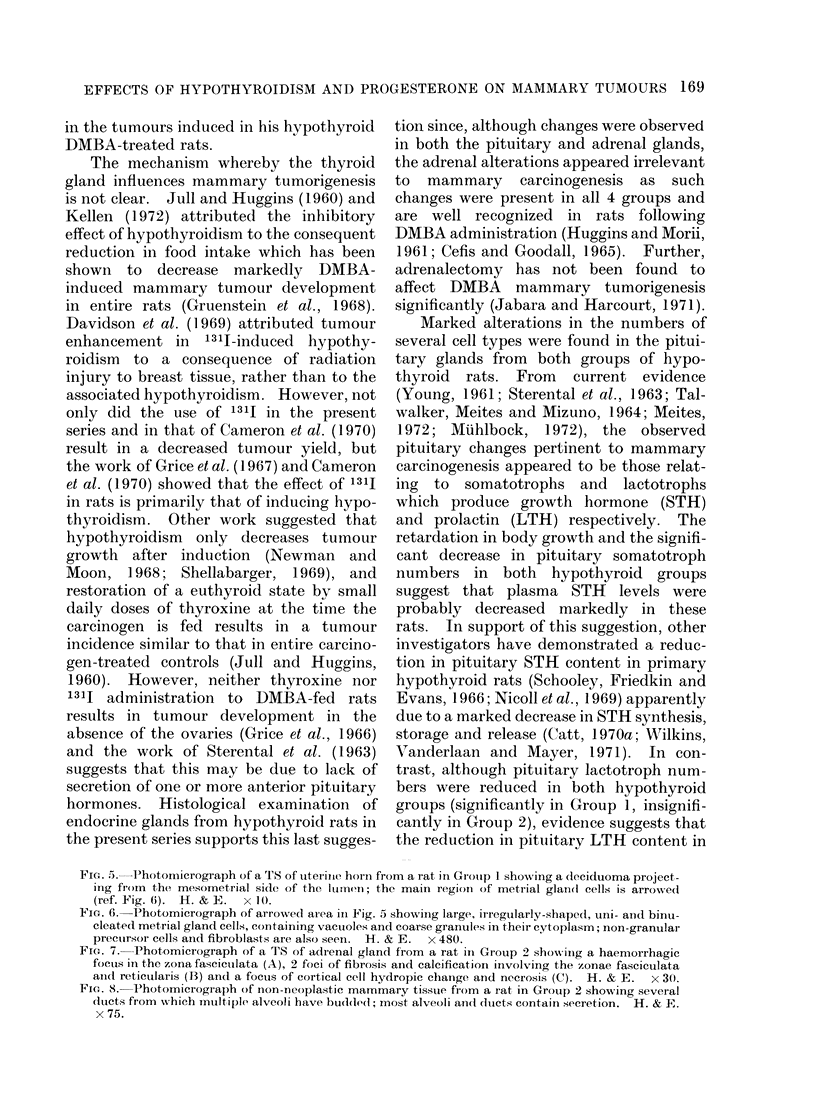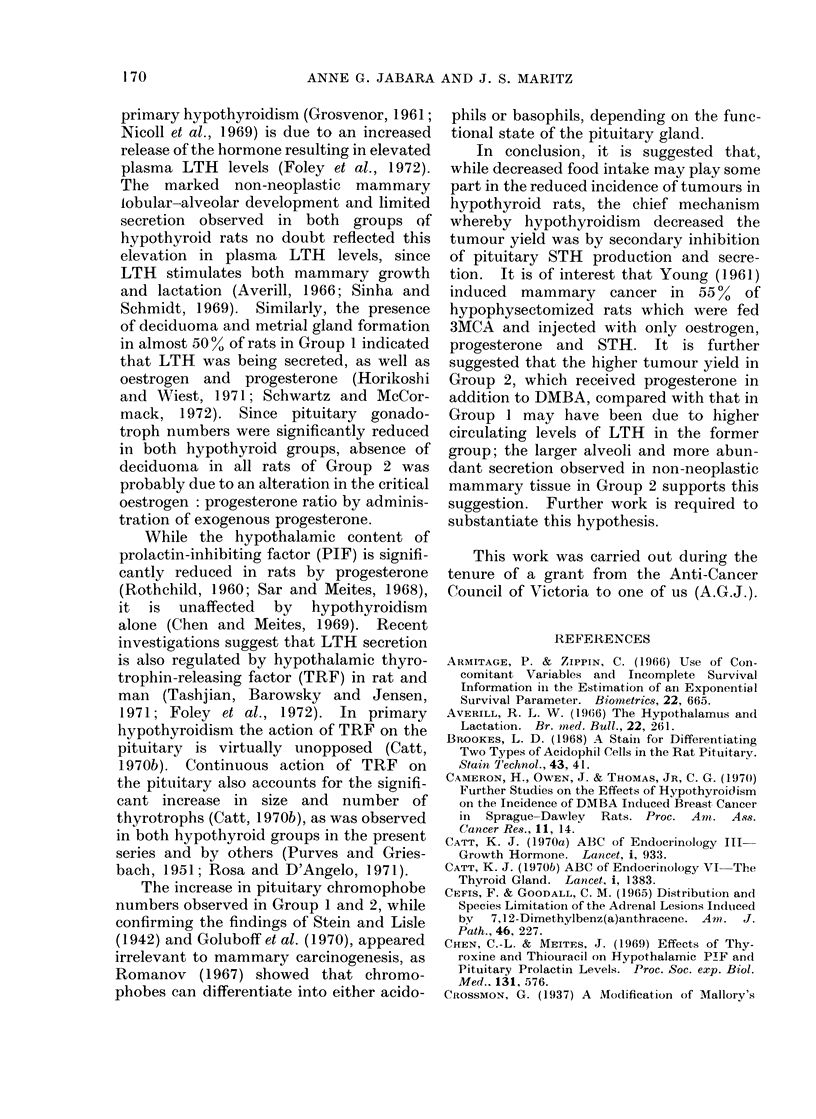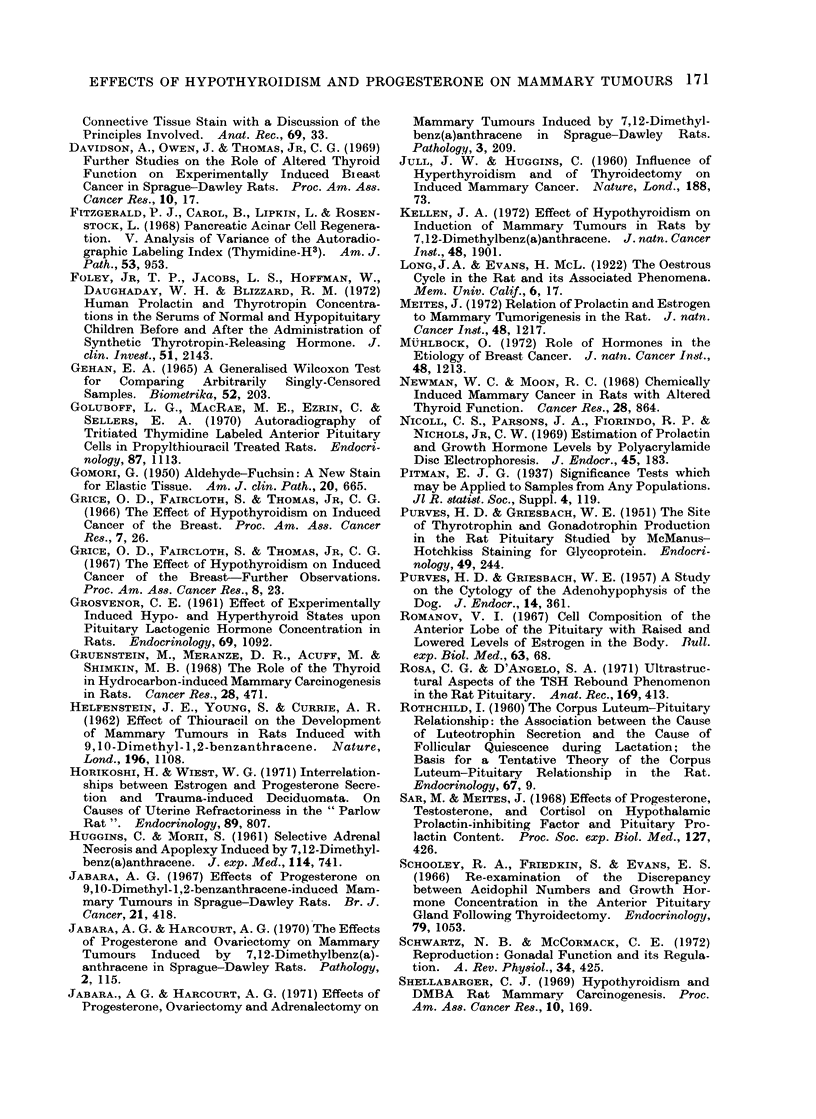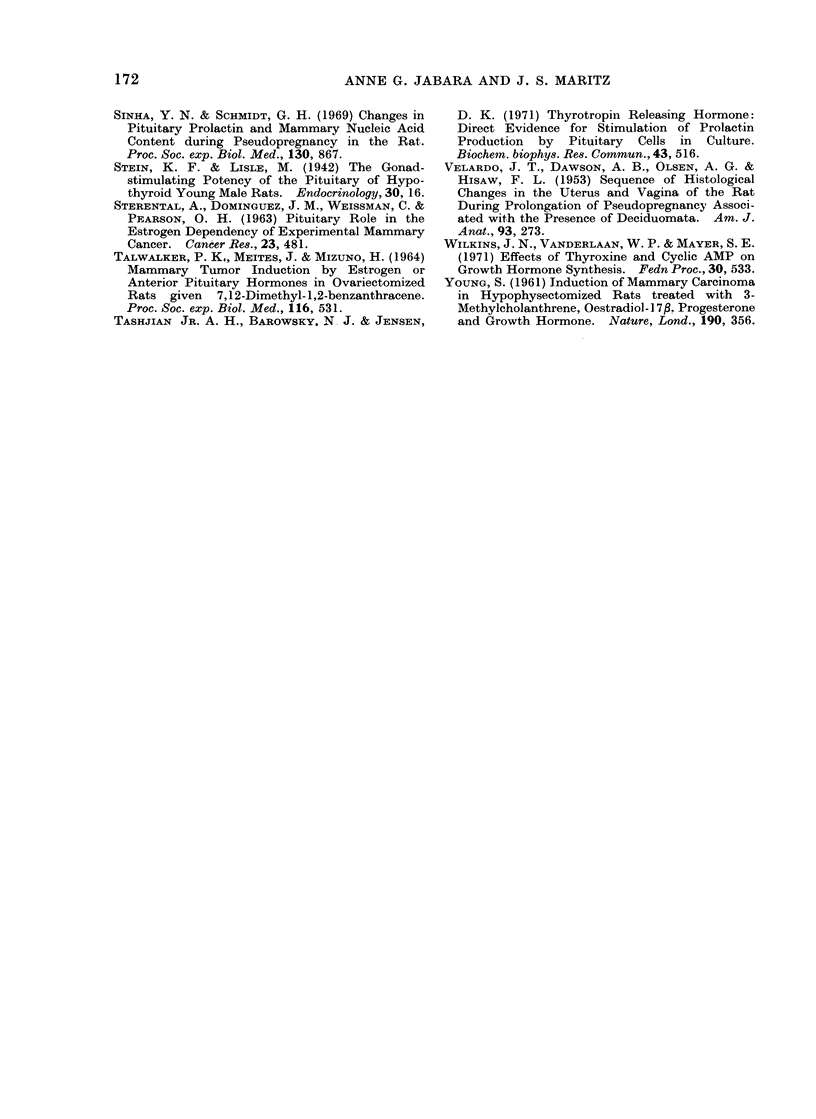# Effects of Hypothyroidism and Progesterone on Mammary Tumours Induced by 7,12-Dimethylbenz(a)anthracene in Sprague-Dawley Rats

**DOI:** 10.1038/bjc.1973.133

**Published:** 1973-08

**Authors:** Anne G. Jabara, J. S. Maritz

## Abstract

**Images:**


*Br. J. Cancer* (1973) **28**, 161

### EFFECTS OF HYPOTHYROIDISM AND PROGESTERONE ON MAMMARY TUMOURS INDUCED BY 7,12-DIMETHYLBENZ(a)ANTHRACENE IN SPRAGUE-DAWLEY RATS

ANNE G. JABARA AND J. S. MARITZ

*From the Departments of Pathology and Statistics, University of Melbourne, Parkville, Victoria, 3052*

Received 29 January 1973. Accepted 9 April 1973

**Summary.—Hypothyroidism, alone or combined with progesterone, significantly decreased 7,12-dimethylbenz(a)anthracene (DMBA) mammary tumorigenesis relative to controls. However, the decrease was less in the progesterone-treated group, and statistical analysis showed that progesterone enhanced tumorigenesis to the same extent in hypothyroid animals as in the controls. Most tumours in hypothyroid progesterone-treated rats were adenocarcinomata; in the absence of the hormone most tumours were benign. However, the difference between the tumour types in the 2 groups was not statistically significant.**

**The morphological changes observed in the endocrine glands, genital tracts and non-neoplastic mammary tissue, considered in relation to previously reported data, suggest that hypothyroidism reduced the tumour yield mainly by secondarily inhibiting somatotrophin production and secretion, although the effect of decreased food intake could not be excluded completely. The higher tumour yield in the hypothyroid progesterone-treated rats may have been due to higher circulating levels of prolactin in this group compared with those in the hypothyroid group which received no hormone.**

INDUCTION of mammary tumours by 7,12-dimethylbenz(a)anthracene (DMBA) is influenced markedly by the hormonal environment of the animal. Progesterone, while not carcinogenic *per se*, significantly enhances DMBA mammary tumorigenesis in entire rats when hormone injections are begun just before or after carcinogen administration (Jabara, 1967; Jabara and Harcourt, 1970). By contrast, ovariectomy performed 7 days before feeding DMBA totally inhibits mammary tumour development (Jabara and Harcourt, 1970) whereas adrenalectomy has no significant effect on tumorigenesis (Jabara and Harcourt, 1971). Reported effects of hypothyroidism on mammary tumour induction range from an enhancement of carcinogenesis (Grice, Faircloth and Thomas, 1966, 1967; Davidson, Owen and Thomas, 1969) to a reduction (Helfenstein, Young and Currie, 1962; Cameron, Owen and Thomas, 1970; Kellen, 1972); Gruenstein *et al.* (1968) found hypothyroidism to be without apparent effect on tumour development. In these reports only tumour incidences and, in some papers, tumour induction times were investigated. As each endocrine gland is part of a functionally related system, histological study of other endocrine glands might help to explain the observed effects of hypothyroidism on DMBA mammary carcinogenesis.

The present experiments were undertaken to determine the effects of hypothyroidism on mammary carcinogenesis and on the endocrine glands, genital tracts and non-neoplastic mammary tissue of rats which had received DMBA, either alone or combined with progesterone.

TABLE I.—*Effects of Hypothyroidism, Alone and Combined with Progesterone, on Mammary Tumour Incidences, Latent Periods, Growth Behaviour and Histological Types Induced after Feeding Rats DMBA, compared with Those Arising in Entire Control Animals*

| Group treatment | Group 1 DMBA + $^{131}I$ | Group 2 DMBA + P+15 + $^{131}I$ | Group 3 DMBA + Sham-ovex | Group 4 DMBA + P + 15 |
|---|---|---|---|---|
| Total no. of rats | 20 | 20 | 20 | 20 |
| Survivors at 4 weeks | 15 | 18 | 19 | 20 |
| Survivors at 28 weeks | 14 | 17 | 11 | 2 |
| No. of rats with tumours | 7 | 11 | 16 | 19 |
| Total no. of tumours | 18 | 27 | 43 | 95 |
| Latent period (days) | (78,78 | (56–186) | (57–188) | (37–86) |
| Average | 96,152) 3 found at autopsy | 112 | 115 | 52 |
| Average No. of active tumour centres per rat | 1·2 | 1·5 | 2·3 | 4·8 |
| Growth behaviour of tumours | | | | |
| No. classified CG | 4 | 6 | 5 | 29 |
| No. classified S | 2 | 11 | 10 | 29 |
| No. classified R | 3 | 3 | 10 | 19 |
| No. unclassified* | 9 | 7 | 18 | 18 |
| Histological tumours types | | | | |
| No. classified carcinoma | 7 | 15 | 23 | 64 |
| No. classified (fibro)adenoma | 11 | 9 | 16 | 23 |
| No. unclassified† | 0 | 3 | 4 | 8 |

* Growth behaviour could not be classified as either no measurements or insufficient measurements of tumours were obtained before death of the host.

† Tumours could not be classified due to complete regression before autopsy.

CG = continuous growth; S = static growth; R = regressing.

### MATERIALS AND METHODS

*Treatment of animals.*—Eighty non-inbred Sprague–Dawley virgin female rats were divided randomly into 4 equal groups (Table I), housed 5 rats/cage, and fed commercial pellets and water *ad libitum.* Rats in Group 3 and 4, which were used as controls in the present experiment (Table I), were previously reported as Group 1 (DMBA + sham-ovex) and Group 3 (DMBA + P+15), respectively (Jabara and Harcourt, 1970). All rats in Group 1 and 2 (Table 1) were injected intraperitoneally with 1 mCi $^{131}I$ as iodide in thiosulphate solution (SA 5 mCi/mmol/l) (Australian Atomic Energy Commission) at 30 ± 1 days of age. At 50 days of age rats in both groups were fed intragastrically a single 30 mg dose of DMBA (Eastman Organic Chemicals, USA) dissolved in 2 ml corn oil. In addition, each rat in Group 2 (DMBA + P+15 + $^{131}I$) received subcutaneous injections of 3 mg of progesterone (Sigma Chemical Co., USA) dissolved in 0·1 ml corn oil/day twice a week for the duration of the experiment (28 weeks); injections were begun on their 65th day of age.

Beginning 4 weeks after DMBA administration, all rats were palpated weekly and any mammary tumours recorded, measured and graphed as described previously (Jabara, 1967).

*Treatment and examination of tissues.*—At autopsy a segment of proximal trachea in the region normally occupied by the thyroid gland was removed, as well as the ovaries, uterus, vagina, adrenals, pituitary gland, mammary tissue and portions of each mammary tumour. Tissues were fixed in 10% buffered formalin and 5 μm paraffin sections were cut from all tissues except the trachea and pituitary gland. Each segment of trachea was serially sectioned, and 2·5–4 μm sections were cut from each pituitary gland in the median transverse plane. All tissue sections other than pituitary gland were stained with haematoxylin and eosin. Pituitary gland sections were stained by Gomori's (1950) aldehyde-fuchsin counter-stained with Crossmon's (1937) modification of Mallory's trichrome, by Brookes' (1968) stain for

TABLE II.—*Histochemical Colour Reactions of Rat Anterior Pituitary Cells and Types of Hormones they Produce*

| Cell type | Trichrome stain | Brookes' stain | PAS/fast green | Type of hormone produced |
|---|---|---|---|---|
| Acidophils | | | | |
| Lactotrophs | Reddish/orange | Red | Pale green | LTH |
| Somatotrophs | Orange | Orange | Strong green | STH |
| Basophils | | | | |
| Gonadotrophs | Purple | Bright green | PAS + | FSH & LH |
| | Pale blue | Bright green | PAS + | |
| Thyrotrophs | Bright blue | Bright green | Strong PAS + | TSH |
| Chromophobes | Weak reddish/ purple | Pale green | Pale green | ?ACTH |

LTH = prolactin; STH = somatotrophin; TSH = thyrotrophin; ACTH = corticotrophin.
FSH = follicular-stimulating hormone } gonadotrophins.
LH = luteinizing hormone differentiating rat acidophils, and by periodic acid–Schiff counterstained with fast green (Purves and Griesbach, 1957) (Table II). Three sections from each anterior pituitary gland were each stained by one of these 3 stains and 300 cells/section counted. A mean differential cell count of 300 cells was recorded for each gland. The fields to be counted in each section were selected by means of the random field method of Fitzgerald *et al.* (1968); use of a 25-square graticule aided in the systematic counting of each field.

*Statistical methods.*—In the statistical analysis of the results, " tumour incidence " was interpreted as the proportion of rats having developed at least one tumour at a time $T$. This is the same as the proportion of rats with a latent period $<T$, and thus an analysis of latent period only was considered adequate as an indicator of differences in tumour incidence. Part of the analysis was based on an exponential model for the distribution of latent periods (Armitage and Zippin, 1966). In this case a change in mean latent period implies a change in tumour incidence and *vice versa*. One difficulty in analysing the data on latent periods was the " censoring " of observations; some rats died before tumours became evident whereas in others tumours were detected only at autopsy. Thus there were lower and upper limits, respectively, to the actual latent period. The latent period data were analysed (1) by adapting the procedure of Armitage and Zippin (1966) which uses a parametric approach and the likelihood ratio technique on the assumption of exponentiallydistributed latent periods, and (2) by Gehan's (1965) distribution-free adaptation for censored data of the Wilcoxon rank sum test. The former procedure was also used to test the significance of main effects and interaction in a multiplicative model for mean latent period, the data being considered as arising from a $2 \times 2$ factorial experiment. The latter method was also used to analyse possible differences in the numbers of tumours developed per rat among different treatment groups. When a rat died before the end of the experiment its number of tumours was taken to be " right " censored in the same way as latent periods were " right " censored. The $\chi^2$-contingency approach was used in the analysis of tumour growth behaviour, whereby tumours were considered as independent entities, and for the detection of possible differences between the proportions of benign to malignant tumours developed among treatment groups. The Pitman (1937) distribution-free test was used to analyse differences in pituitary cell counts.

### RESULTS

No mammary tumour was observed during the first 4 weeks following carcinogen administration. During this period, 5 rats in Group 1 and 2 rats in Group 2 died from the toxic effects of DMBA between 2 and 19 days after receiving the carcinogen (Table I). In addition, 1 rat died in each of Group 1 and 2 at 55 and 46 days respectively, the remaining animals all surviving to the end of the experiment (28 weeks after feeding DMBA) (Table I).

FIG. 1

FIG. 3

FIG. 2

FIG. 4

*Evidence of hypothyroidism*

A pilot study showed that 1 mCi of $^{131}I$ given to 30-day old rats produced marked thyroid atrophy within 14–20 days; hence rats in Group 1 and 2 should have been hypothyroid when DMBA was administered at 50 days of age (20 days after $^{131}I$ administration), an assumption borne out by the following findings: (1) Serial sections of the proximal trachea at the termination of the experiment showed that the thyroid gland was markedly atrophic in every rat of Group 1 and 2, although the extent of atrophy varied. In most rats both lobes were either completely or partially fibrosed, the fibrous tissue sometimes showing hyaline degeneration and frequently calcification with an accompanying foreign body inflammatory reaction (Fig. 1–3). Where fibrosis was incomplete, very small follicles remained whose lumina were generally empty (Fig. 2 and 4). Likewise, the isthmus in all animals was atrophic and was composed of very small follicles, frequently devoid of colloid (Fig. 1). In all rats the parathyroids either appeared normal or showed slight atrophy and minimal fibrosis (Fig. 4). (2) Large numbers of thyroidectomy cells were present in the anterior pituitary glands from rats in both groups (1 and 2) (see below). (3) Each animal in Group 1 and 2 had a dry, scaly skin bearing sparse brittle hair. (4) There was marked stunting of body growth. The mean body weights of the rats at the conclusion of the experiment were 182 and 204 g in Group 1 and 2 respectively, compared with the control means of 273 and 306 g in Group 3 and 4 respectively.

*Tumour incidence and latent period*

The findings are shown in Table I. Both methods of statistical analysis of these data gave substantially similar results, and the $P$ values reported below are those obtained by Gehan's (1965) method.

Mammary tumours arose in both groups of hypothyroid rats. Comparison of the latent periods (tumour incidences) in Group 1 and 3 showed that hypothyroidism caused a significant lengthening of tumour induction time ($P < 0{\cdot}02$). Similarly, the average latent period in Group 2, while not significantly different from that in Group 1 or 3, was significantly longer than that in Group 4 ($P < 0{\cdot}001$).

A $2 \times 2$ factorial analysis revealed that, while the main effect of $^{131}I$ was significant ($P < 0{\cdot}001$) and the main effect of progesterone was significant ($P < 0{\cdot}05$), there was no significant interaction between $^{131}I$ and progesterone in relation to the latent period in all 4 groups of rats ($P$ approximated 0·30).

*Active tumour centres*

Hypothyroidism significantly reduced the average number of active tumour centres per rat in Group 1 and 2 relative to Group 3, whether or not progesterone was also administered ($P < 0{\cdot}005$ and $P < 0{\cdot}01$ respectively) (Table I). Although the reduction was less marked

FIG. 1.—Photomicrograph of a TS of thyroid gland and trachea from a rat in Group 2 (DMBA + $P + 15$ + $^{131}I$) showing complete fibrosis of one lobe and almost complete fibrosis of the other except for a small group of tiny, empty follicles (arrowed) (ref. Fig. 2). The isthmus is atrophic and composed of very small, mostly empty, follicles. H. & E. ×30.

FIG. 2.—Higher-power TS view of an almost totally fibrosed thyroid lobe showing hyalinization of the stroma and the presence of a few very small follicles, one of which contains colloid. H. & E. ×75.

FIG. 3.—Photomicrograph of a TS of a fibrosed thyroid lobe showing an area of calcification accompanied by a foreign body inflammatory reaction (arrows indicate 2 multinucleated giant cells). H. & E. ×75.

FIG. 4.—Photomicrograph of a TS of the least atrophic thyroid lobe found in the series which was derived from a rat in Group 1 (DMBA + $^{131}I$). Note the presence of multiple small follicles, some of which contain a little colloid, the increase in fibrous tissue and the normal parathyroid gland. H. & E. ×75.

in Group 2 than in Group 1, the difference was not significant.

*Tumour growth behaviour*

Three main types of tumour growth behaviour (Jabara, 1967) occurred in both groups of hypothyroid animals (Table I). Only 9 tumours in Group 1 and 20 tumours in Group 2 could be followed long enough to assess their growth behaviour. In Group 1, similar numbers of tumours showed either continuous growth or partial regression, while in Group 2 more than half the neoplasms remained static after an initial short growth period during which, with the exception of one tumour, they became palpable but did not reach a measurable size (1 cm or more in longest diameter) (Table I). However, differences between the numbers of classifiable neoplasms exhibiting the 3 main types of tumour growth in the 2 hypothyroid groups were not statistically significant, indicating that the presence of progesterone (Group 2) did not modify tumour growth behaviour.

No direct correlation was apparent between growth behaviour and histological tumour type in Group 1 and 2, or between growth behaviour and microscopic structure within a particular neoplastic type.

*Locations and types of tumours*

As has been observed previously in entire rats (Jabara and Harcourt, 1970), in Group 1 and 2 neoplasms arose equally on both sides and most tumours were in the anterior 3 pairs of glands.

All 3 histological types of tumours (Jabara, 1967) occurred in Group 1 and 2. In Group 1 most tumours were benign (11/18), while in Group 2 the majority were malignant (15/24), most being adenocarcinomata (Table I). However, the difference between the tumour types in the 2 groups was not statistically significant.

*Ovaries, uteri and vaginae*

Microscopic examination of the ovaries, uteri and vaginae from the hypothyroid rats in Group 1 revealed that all animals at death were either in dioestrus or were pseudopregnant (Long and Evans, 1922; Velardo *et al.*, 1953). The latter diagnosis was supported by the presence of deciduomata (Velardo *et al.*, 1953) in one or both uterine cornu in 47% (7/15) of animals (Fig. 5 and 6). Of these 7 rats, 4 bore 1 or more mammary neoplasms and 3 were tumour free. Hence, there was no apparent correlation between deciduoma formation and tumour development.

In Group 2, 72% (13/18) of rats were in dioestrus, 17% (3/18) in pro-oestrus and 11% (2/18) in oestrus. No deciduoma was observed in the uterus of any rat in this group.

*Adrenal glands*

The adrenals derived from animals in Group 1 and 2 were histologically similar and appeared slightly atrophic compared with those from the control groups. However, the degree of adrenal cortical necrosis, haemorrhage and calcification (Fig. 7) was comparable in all 4 groups.

*Anterior pituitary glands*

In comparison with differential cell counts of anterior pituitary glands from 22 untreated entire rats (Table II and III), hypothyroidism in Group 1 significantly reduced the number of lactotrophs ($P < 0{\cdot}001$) and somatotrophs ($P < 0{\cdot}001$) and, further, both types of acidophils showed a marked loss of cytoplasmic granules. There were also significantly fewer gonadotrophs in Group 1 ($P < 0{\cdot}001$), while both the thyrotrophs and chromophobes were increased significantly ($P < 0{\cdot}001$ and $< 0{\cdot}001$ respectively) (Table II and III); the thyrotrophs were also markedly enlarged, vacuolated and pleomorphic, and corresponded to thyroidectomy cells (Purves and Griesbach, 1951, 1957). With the exception of the lactotrophs, similar significant alterations in differential cell counts were observed in Group 2 pituitary glands as were seen

TABLE III.—*Treatments Used and the Resulting Mean Counts and Standard Errors of Pituitary Cell Types per 300 Cells per Gland compared with those in Pituitaries from Untreated Control Rats*

| Pituitary cell type | Untreated controls | DMBA + $^{131}I$ | DMBA + P+15 + $^{131}I$ |
|---|---|---|---|
| Lactotrophs | 28·5 ± 0·65 | 11·3 ± 1·20* | 23·5 ± 3·33 |
| Somatotrophs | 78·3 ± 1·89 | 3·3 ± 0·67* | 3·5 ± 0·56* |
| Gonadotrophs | 21·8 ± 0·76 | 10·7 ± 2·03* | 11·2 ± 0·99* |
| Thyrotrophs | 6·0 ± 0·46 | 77·0 ± 5·57* | 68·2 ± 4·82* |
| Chromophobes | 165·4 ± 2·78 | 197·7 ± 4·70* | 193·6 ± 5·33* |

* Difference between a cell type number in a treatment group and that in untreated controls was significant ($P < 0·001$).

in those from Group 1 (Table III). In Group 2, however, the lactotrophs were only insignificantly reduced compared with their numbers in pituitaries from the untreated controls, and just failed at the 5% level to be significantly increased compared with the number in pituitary glands from rats in Group 1 ($P = 0·054$) (Table III).

*Mammary tissue (non-neoplastic)*

A subjective histological assessment of non-neoplastic mammary lobular–alveolar development in the 4 groups of rats revealed that it was approximately comparable in both hypothyroid groups, although mammae in Group 2 contained slightly larger alveoli and more secretion than did those in Group 1 (Fig. 8). Such development was much greater in these 2 groups than that seen in either of the control groups; in the 2 control groups glandular development was less obvious in the mammae derived from animals treated with only DMBA.

### DISCUSSION

The observation that hypothyroidism decreased, but did not totally inhibit, DMBA tumorigenesis confirms the findings of Helfenstein *et al.* (1962), Cameron *et al.* (1970) and Kellen (1972) for DMBA tumorigenesis, and Jull and Huggins (1960) and Newman and Moon (1968) for mammary tumours induced by 3-methylcholanthrene (3MCA). Grice *et al.* (1966, 1967) and Davidson *et al.* (1969) claimed that hypothyroidism increased the incidence of DMBA-induced mammary tumours. However, these reports are difficult to evaluate because the strain of rat used by Davidson and colleagues was not stated and Grice *et al.* (1966, 1967) did not record the dose of $^{131}I$ used or the time of its administration to Sprague–Dawley rats; the time at which hypothyroidism is induced in relation to the time of feeding DMBA has been shown to affect profoundly the resulting tumour incidence (Shellabarger, 1969).

Continuous progesterone administration to hypothyroid rats increased DMBA tumorigenesis compared with hypothyroidism alone, although the tumour yield was still significantly lower and the latent period significantly longer than in the control groups treated with DMBA, either alone or combined with progesterone (Jabara and Harcourt, 1970). However, statistically the enhancing effect of progesterone on latent period (tumour incidence) in hypothyroid rats was equivalent to that previously observed in entire DMBA-treated animals (Jabara, 1967; Jabara and Harcourt, 1970).

Progesterone administration did not modify tumour growth behaviour in hypothyroid rats; a similar finding was observed previously when progesterone was given to the controls (Jabara and Harcourt, 1970).

More tumours induced by DMBA in the hypothyroid rats of Group 1 were benign than in any of the other 3 groups, but this difference was not statistically significant. Kellen (1972) reported a similar shift towards a more benign histological pattern

Fig. 5

Fig. 7

Fig. 6

Fig. 8

in the tumours induced in his hypothyroid DMBA-treated rats.

The mechanism whereby the thyroid gland influences mammary tumorigenesis is not clear. Jull and Huggins (1960) and Kellen (1972) attributed the inhibitory effect of hypothyroidism to the consequent reduction in food intake which has been shown to decrease markedly DMBA-induced mammary tumour development in entire rats (Gruenstein *et al.*, 1968). Davidson *et al.* (1969) attributed tumour enhancement in $^{131}$I-induced hypothyroidism to a consequence of radiation injury to breast tissue, rather than to the associated hypothyroidism. However, not only did the use of $^{131}$I in the present series and in that of Cameron *et al.* (1970) result in a decreased tumour yield, but the work of Grice *et al.* (1967) and Cameron *et al.* (1970) showed that the effect of $^{131}$I in rats is primarily that of inducing hypothyroidism. Other work suggested that hypothyroidism only decreases tumour growth after induction (Newman and Moon, 1968; Shellabarger, 1969), and restoration of a euthyroid state by small daily doses of thyroxine at the time the carcinogen is fed results in a tumour incidence similar to that in entire carcinogen-treated controls (Jull and Huggins, 1960). However, neither thyroxine nor $^{131}$I administration to DMBA-fed rats results in tumour development in the absence of the ovaries (Grice *et al.*, 1966) and the work of Sterental *et al.* (1963) suggests that this may be due to lack of secretion of one or more anterior pituitary hormones. Histological examination of endocrine glands from hypothyroid rats in the present series supports this last suggestion since, although changes were observed in both the pituitary and adrenal glands, the adrenal alterations appeared irrelevant to mammary carcinogenesis as such changes were present in all 4 groups and are well recognized in rats following DMBA administration (Huggins and Morii, 1961; Cefis and Goodall, 1965). Further, adrenalectomy has not been found to affect DMBA mammary tumorigenesis significantly (Jabara and Harcourt, 1971).

Marked alterations in the numbers of several cell types were found in the pituitary glands from both groups of hypothyroid rats. From current evidence (Young, 1961; Sterental *et al.*, 1963; Talwalker, Meites and Mizuno, 1964; Meites, 1972; Mühlbock, 1972), the observed pituitary changes pertinent to mammary carcinogenesis appeared to be those relating to somatotrophs and lactotrophs which produce growth hormone (STH) and prolactin (LTH) respectively. The retardation in body growth and the significant decrease in pituitary somatotroph numbers in both hypothyroid groups suggest that plasma STH levels were probably decreased markedly in these rats. In support of this suggestion, other investigators have demonstrated a reduction in pituitary STH content in primary hypothyroid rats (Schooley, Friedkin and Evans, 1966; Nicoll *et al.*, 1969) apparently due to a marked decrease in STH synthesis, storage and release (Catt, 1970*a*; Wilkins, Vanderlaan and Mayer, 1971). In contrast, although pituitary lactotroph numbers were reduced in both hypothyroid groups (significantly in Group 1, insignificantly in Group 2), evidence suggests that the reduction in pituitary LTH content in

FIG. 5.—Photomicrograph of a TS of uterine horn from a rat in Group 1 showing a deciduoma projecting from the mesometrial side of the lumen; the main region of metrial gland cells is arrowed (ref. Fig. 6). H. & E. ×10.

FIG. 6.—Photomicrograph of arrowed area in Fig. 5 showing large, irregularly-shaped, uni- and binucleated metrial gland cells, containing vacuoles and coarse granules in their cytoplasm; non-granular precursor cells and fibroblasts are also seen. H. & E. ×480.

FIG. 7.—Photomicrograph of a TS of adrenal gland from a rat in Group 2 showing a haemorrhagic focus in the zona fasciculata (A), 2 foci of fibrosis and calcification involving the zonae fasciculata and reticularis (B) and a focus of cortical cell hydropic change and necrosis (C). H. & E. ×30.

FIG. 8.—Photomicrograph of non-neoplastic mammary tissue from a rat in Group 2 showing several ducts from which multiple alveoli have budded; most alveoli and ducts contain secretion. H. & E. ×75.

primary hypothyroidism (Grosvenor, 1961; Nicoll *et al.*, 1969) is due to an increased release of the hormone resulting in elevated plasma LTH levels (Foley *et al.*, 1972). The marked non-neoplastic mammary lobular–alveolar development and limited secretion observed in both groups of hypothyroid rats no doubt reflected this elevation in plasma LTH levels, since LTH stimulates both mammary growth and lactation (Averill, 1966; Sinha and Schmidt, 1969). Similarly, the presence of deciduoma and metrial gland formation in almost 50% of rats in Group 1 indicated that LTH was being secreted, as well as oestrogen and progesterone (Horikoshi and Wiest, 1971; Schwartz and McCormack, 1972). Since pituitary gonadotroph numbers were significantly reduced in both hypothyroid groups, absence of deciduoma in all rats of Group 2 was probably due to an alteration in the critical oestrogen : progesterone ratio by administration of exogenous progesterone.

While the hypothalamic content of prolactin-inhibiting factor (PIF) is significantly reduced in rats by progesterone (Rothchild, 1960; Sar and Meites, 1968), it is unaffected by hypothyroidism alone (Chen and Meites, 1969). Recent investigations suggest that LTH secretion is also regulated by hypothalamic thyrotrophin-releasing factor (TRF) in rat and man (Tashjian, Barowsky and Jensen, 1971; Foley *et al.*, 1972). In primary hypothyroidism the action of TRF on the pituitary is virtually unopposed (Catt, 1970*b*). Continuous action of TRF on the pituitary also accounts for the significant increase in size and number of thyrotrophs (Catt, 1970*b*), as was observed in both hypothyroid groups in the present series and by others (Purves and Griesbach, 1951; Rosa and D'Angelo, 1971).

The increase in pituitary chromophobe numbers observed in Group 1 and 2, while confirming the findings of Stein and Lisle (1942) and Goluboff *et al.* (1970), appeared irrelevant to mammary carcinogenesis, as Romanov (1967) showed that chromophobes can differentiate into either acidophils or basophils, depending on the functional state of the pituitary gland.

In conclusion, it is suggested that, while decreased food intake may play some part in the reduced incidence of tumours in hypothyroid rats, the chief mechanism whereby hypothyroidism decreased the tumour yield was by secondary inhibition of pituitary STH production and secretion. It is of interest that Young (1961) induced mammary cancer in 55% of hypophysectomized rats which were fed 3MCA and injected with only oestrogen, progesterone and STH. It is further suggested that the higher tumour yield in Group 2, which received progesterone in addition to DMBA, compared with that in Group 1 may have been due to higher circulating levels of LTH in the former group; the larger alveoli and more abundant secretion observed in non-neoplastic mammary tissue in Group 2 supports this suggestion. Further work is required to substantiate this hypothesis.

This work was carried out during the tenure of a grant from the Anti-Cancer Council of Victoria to one of us (A.G.J.).

### REFERENCES

ARMITAGE, P. & ZIPPIN, C. (1966) Use of Concomitant Variables and Incomplete Survival Information in the Estimation of an Exponential Survival Parameter. *Biometrics*, **22**, 665.

AVERILL, R. L. W. (1966) The Hypothalamus and Lactation. *Br. med. Bull.*, **22**, 261.

BROOKES, L. D. (1968) A Stain for Differentiating Two Types of Acidophil Cells in the Rat Pituitary. *Stain Technol.*, **43**, 41.

CAMERON, H., OWEN, J. & THOMAS, JR, C. G. (1970) Further Studies on the Effects of Hypothyroidism on the Incidence of DMBA Induced Breast Cancer in Sprague–Dawley Rats. *Proc. Am. Ass. Cancer Res.*, **11**, 14.

CATT, K. J. (1970*a*) ABC of Endocrinology III—Growth Hormone. *Lancet*, **i**, 933.

CATT, K. J. (1970*b*) ABC of Endocrinology VI—The Thyroid Gland. *Lancet*, **i**, 1383.

CEFIS, F. & GOODALL, C. M. (1965) Distribution and Species Limitation of the Adrenal Lesions Induced by 7,12-Dimethylbenz(a)anthracene. *Am. J. Path.*, **46**, 227.

CHEN, C.-L. & MEITES, J. (1969) Effects of Thyroxine and Thiouracil on Hypothalamic PIF and Pituitary Prolactin Levels. *Proc. Soc. exp. Biol. Med.*, **131**, 576.

CROSSMON, G. (1937) A Modification of Mallory's

Connective Tissue Stain with a Discussion of the Principles Involved. *Anat. Rec.*, **69**, 33.

Davidson, A., Owen, J. & Thomas, Jr, C. G. (1969) Further Studies on the Role of Altered Thyroid Function on Experimentally Induced Bieast Cancer in Sprague–Dawley Rats. *Proc. Am. Ass. Cancer Res.*, **10**, 17.

Fitzgerald, P. J., Carol, B., Lipkin, L. & Rosenstock, L. (1968) Pancreatic Acinar Cell Regeneration. V. Analysis of Variance of the Autoradiographic Labeling Index (Thymidine-$H^3$). *Am. J. Path.*, **53**, 953.

Foley, Jr, T. P., Jacobs, L. S., Hoffman, W., Daughaday, W. H. & Blizzard, R. M. (1972) Human Prolactin and Thyrotropin Concentrations in the Serums of Normal and Hypopituitary Children Before and After the Administration of Synthetic Thyrotropin-Releasing Hormone. *J. clin. Invest.*, **51**, 2143.

Gehan, E. A. (1965) A Generalised Wilcoxon Test for Comparing Arbitrarily Singly-Censored Samples. *Biometrika*, **52**, 203.

Goluboff, L. G., MacRae, M. E., Ezrin, C. & Sellers, E. A. (1970) Autoradiography of Tritiated Thymidine Labeled Anterior Pituitary Cells in Propylthiouracil Treated Rats. *Endocrinology*, **87**, 1113.

Gomori, G. (1950) Aldehyde–Fuchsin: A New Stain for Elastic Tissue. *Am. J. clin. Path.*, **20**, 665.

Grice, O. D., Faircloth, S. & Thomas, Jr, C. G. (1966) The Effect of Hypothyroidism on Induced Cancer of the Breast. *Proc. Am. Ass. Cancer Res.*, **7**, 26.

Grice, O. D., Faircloth, S. & Thomas, Jr, C. G. (1967) The Effect of Hypothyroidism on Induced Cancer of the Breast—Further Observations. *Proc. Am. Ass. Cancer Res.*, **8**, 23.

Grosvenor, C. E. (1961) Effect of Experimentally Induced Hypo- and Hyperthyroid States upon Pituitary Lactogenic Hormone Concentration in Rats. *Endocrinology*, **69**, 1092.

Gruenstein, M., Meranze, D. R., Acuff, M. & Shimkin, M. B. (1968) The Role of the Thyroid in Hydrocarbon-induced Mammary Carcinogenesis in Rats. *Cancer Res.*, **28**, 471.

Helfenstein, J. E., Young, S. & Currie, A. R. (1962) Effect of Thiouracil on the Development of Mammary Tumours in Rats Induced with 9,10-Dimethyl-1,2-benzanthracene. *Nature, Lond.*, **196**, 1108.

Horikoshi, H. & Wiest, W. G. (1971) Interrelationships between Estrogen and Progesterone Secretion and Trauma-induced Deciduomata. On Causes of Uterine Refractoriness in the " Parlow Rat ". *Endocrinology*, **89**, 807.

Huggins, C. & Morii, S. (1961) Selective Adrenal Necrosis and Apoplexy Induced by 7,12-Dimethylbenz(a)anthracene. *J. exp. Med.*, **114**, 741.

Jabara, A. G. (1967) Effects of Progesterone on 9,10-Dimethyl-1,2-benzanthracene-induced Mammary Tumours in Sprague–Dawley Rats. *Br. J. Cancer*, **21**, 418.

Jabara, A. G. & Harcourt, A. G. (1970) The Effects of Progesterone and Ovariectomy on Mammary Tumours Induced by 7,12-Dimethylbenz(a)-anthracene in Sprague–Dawley Rats. *Pathology*, **2**, 115.

Jabara., A G. & Harcourt, A. G. (1971) Effects of Progesterone, Ovariectomy and Adrenalectomy on Mammary Tumours Induced by 7,12-Dimethylbenz(a)anthracene in Sprague–Dawley Rats. *Pathology*, **3**, 209.

Jull, J. W. & Huggins, C. (1960) Influence of Hyperthyroidism and of Thyroidectomy on Induced Mammary Cancer. *Nature, Lond.*, **188**, 73.

Kellen, J. A. (1972) Effect of Hypothyroidism on Induction of Mammary Tumours in Rats by 7,12-Dimethylbenz(a)anthracene. *J. natn. Cancer Inst.*, **48**, 1901.

Long, J. A. & Evans, H. McL. (1922) The Oestrous Cycle in the Rat and its Associated Phenomena. *Mem. Univ. Calif.*, **6**, 17.

Meites, J. (1972) Relation of Prolactin and Estrogen to Mammary Tumorigenesis in the Rat. *J. natn. Cancer Inst.*, **48**, 1217.

Mühlbock, O. (1972) Role of Hormones in the Etiology of Breast Cancer. *J. natn. Cancer Inst.*, **48**, 1213.

Newman, W. C. & Moon, R. C. (1968) Chemically Induced Mammary Cancer in Rats with Altered Thyroid Function. *Cancer Res.*, **28**, 864.

Nicoll, C. S., Parsons, J. A., Fiorindo, R. P. & Nichols, Jr, C. W. (1969) Estimation of Prolactin and Growth Hormone Levels by Polyacrylamide Disc Electrophoresis. *J. Endocr.*, **45**, 183.

Pitman, E. J. G. (1937) Significance Tests which may be Applied to Samples from Any Populations. *Jl R. statist. Soc.*, Suppl. **4**, 119.

Purves, H. D. & Griesbach, W. E. (1951) The Site of Thyrotrophin and Gonadotrophin Production in the Rat Pituitary Studied by McManus–Hotchkiss Staining for Glycoprotein. *Endocrinology*, **49**, 244.

Purves, H. D. & Griesbach, W. E. (1957) A Study on the Cytology of the Adenohypophysis of the Dog. *J. Endocr.*, **14**, 361.

Romanov, V. I. (1967) Cell Composition of the Anterior Lobe of the Pituitary with Raised and Lowered Levels of Estrogen in the Body. *Bull. exp. Biol. Med.*, **63**, 68.

Rosa, C. G. & D'Angelo, S. A. (1971) Ultrastructural Aspects of the TSH Rebound Phenomenon in the Rat Pituitary. *Anat. Rec.*, **169**, 413.

Rothchild, I. (1960) The Corpus Luteum–Pituitary Relationship: the Association between the Cause of Luteotrophin Secretion and the Cause of Follicular Quiescence during Lactation; the Basis for a Tentative Theory of the Corpus Luteum–Pituitary Relationship in the Rat. *Endocrinology*, **67**, 9.

Sar, M. & Meites, J. (1968) Effects of Progesterone, Testosterone, and Cortisol on Hypothalamic Prolactin-inhibiting Factor and Pituitary Prolactin Content. *Proc. Soc. exp. Biol. Med.*, **127**, 426.

Schooley, R. A., Friedkin, S. & Evans, E. S. (1966) Re-examination of the Discrepancy between Acidophil Numbers and Growth Hormone Concentration in the Anterior Pituitary Gland Following Thyroidectomy. *Endocrinology*, **79**, 1053.

Schwartz, N. B. & McCormack, C. E. (1972) Reproduction: Gonadal Function and its Regulation. *A. Rev. Physiol.*, **34**, 425.

Shellabarger, C. J. (1969) Hypothyroidism and DMBA Rat Mammary Carcinogenesis. *Proc. Am. Ass. Cancer Res.*, **10**, 169.

SINHA, Y. N. & SCHMIDT, G. H. (1969) Changes in Pituitary Prolactin and Mammary Nucleic Acid Content during Pseudopregnancy in the Rat. *Proc. Soc. exp. Biol. Med.*, **130**, 867.

STEIN, K. F. & LISLE, M. (1942) The Gonad-stimulating Potency of the Pituitary of Hypothyroid Young Male Rats. *Endocrinology*, **30**, 16.

STERENTAL, A., DOMINGUEZ, J. M., WEISSMAN, C. & PEARSON, O. H. (1963) Pituitary Role in the Estrogen Dependency of Experimental Mammary Cancer. *Cancer Res.*, **23**, 481.

TALWALKER, P. K., MEITES, J. & MIZUNO, H. (1964) Mammary Tumor Induction by Estrogen or Anterior Pituitary Hormones in Ovariectomized Rats given 7,12-Dimethyl-1,2-benzanthracene. *Proc. Soc. exp. Biol. Med.*, **116**, 531.

TASHJIAN JR. A. H., BAROWSKY, N. J. & JENSEN, D. K. (1971) Thyrotropin Releasing Hormone: Direct Evidence for Stimulation of Prolactin Production by Pituitary Cells in Culture. *Biochem. biophys. Res. Commun.*, **43**, 516.

VELARDO, J. T., DAWSON, A. B., OLSEN, A. G. & HISAW, F. L. (1953) Sequence of Histological Changes in the Uterus and Vagina of the Rat During Prolongation of Pseudopregnancy Associated with the Presence of Deciduomata. *Am. J. Anat.*, **93**, 273.

WILKINS, J. N., VANDERLAAN, W. P. & MAYER, S. E. (1971) Effects of Thyroxine and Cyclic AMP on Growth Hormone Synthesis. *Fedn Proc.*, **30**, 533.

YOUNG, S. (1961) Induction of Mammary Carcinoma in Hypophysectomized Rats treated with 3-Methylcholanthrene, Oestradiol-17$\beta$, Progesterone and Growth Hormone. *Nature, Lond.*, **190**, 356.